# Antipredator responses of bats during short boreal nights with variable climatic conditions

**DOI:** 10.1093/jmammal/gyae124

**Published:** 2024-10-29

**Authors:** Miina S Suutari, Aleksi Lehikoinen, Harry J Lehto, Thomas M Lilley

**Affiliations:** Finnish Museum of Natural History, University of Helsinki, 00014 Helsinki, Finland; Finnish Museum of Natural History, University of Helsinki, 00014 Helsinki, Finland; Tuorla Observatory, University of Turku, 20014 Turku, Finland; Finnish Museum of Natural History, University of Helsinki, 00014 Helsinki, Finland

**Keywords:** acoustic cues, antipredator responses, boreal zone, Chiroptera, predation

## Abstract

The threat of predation can influence the behavior of animals. To minimize the impact of predation, animals rely on antipredatory responses and effectively balance these responses with other activities to maximize survival. Boreal bats are nocturnal animals that must forage within a narrow time frame during short, light summer nights with unpredictable weather. Despite having no specialized predators, boreal bats are still subject to predation. However, whether they express antipredatory responses has not been established. We studied antipredatory responses and responses to climatic conditions in boreal bats in 2 settings: 1) during roost emergence; and 2) during foraging within Tawny Owl territories and at locations with no Tawny Owl sightings. Acoustic data were collected at 23 roosts and 10 foraging grounds. Two controlled predation threats were used—playbacks of Tawny Owl calls and fledgling calls. Fledgling calls were only played during roost emergence. In both experiments, music and no treatment were used as controls. We also incorporated weather variables in the model. According to our results, bats tend to delay their emergence by 16 min when Tawny Owl calls were played outside the roost, but this effect was not noticeable when weather variables were included. There was no difference in exit time when music or fledgling sounds were played. While foraging, bats reduced their activity in Tawny Owl territories when calls of owls or music were played compared to no treatment. These results suggest that bats might display variable antipredatory responses, but weather influences behavior of bats more than predation risk, highlighting the importance of energy-saving strategies at northerly latitudes.

## Teaser

Antipredator behavior should increase fitness of animals but bats present mixed signals. Bat emergence was delayed while under simulated threat but was overpowered by responses to weather. While foraging within owl territories bats decreased their activity while under any treatment.

Predation has power to influence animal behavior from the level of an individual to the dynamics of a population (Pettorelli et al. 2011). The pressure caused by predation can be severe and has the potential to cause major mortality in a population (Angelstam et al. 1984). In addition, predation risk can influence behavior and decision-making in animals, such as the timing and location of foraging, or the activity levels and active periods of prey (Lind and Cresswell 2005; Creel and Christianson 2008; Laundré et al. 2010). Because a successful predation event is fatal, any antipredatory trait that facilitates escape from an attack or allows the animal to avoid an attack altogether has the potential to provide an advantage in the form of increased fitness (Lind and Cresswell 2005).

Bats (Chiroptera, Vespertilionidae) are the second largest order of mammals (Burgin et al. 2018), but studies on their antipredatory responses are still quite rare. Some traits associated with bats—including nocturnality, clustered roost emergence, and roosting in large groups—have been proposed as antipredatory behavior (Fenton and Fleming 1976; Jones and Rydell 1994; Duvergé et al. 2000; Lima and O’Keefe 2013), but there has been no consensus on whether they are directly connected to predator avoidance. Nocturnality is an almost universal trait for bats, with regularly day-active species only found on remote islands with no diurnal raptors (Russo et al. 2011). Otherwise, diurnal activity of bats is hypothesized to be linked to environmental or physiological stressors, such as poor nocturnal foraging conditions (Speakman 1990), malnourishment, or dehydration (Speakman 1990; Jung 2013; Bôlla et al. 2017). Indeed, bats are much more vulnerable to predation in daylight (Mikula et al. 2016), and tropics bats are known to even avoid strong moonlight (lunar phobia) as an antipredatory response (Lima and O’Keefe 2013; Saldaña-Vázquez and Munguía-Rosas 2013; Roeleke et al. 2018). However, in the boreal zone, nocturnal animals may not be able to operate only in darkness as the seasonal light conditions vary greatly, with summer nights being short and dimly light. Thus, nocturnal species have limited time for foraging and must adjust their behavior to these conditions (Boyles et al. 2016; Eriksen and Wabakken 2018). For instance, bats at high northern latitudes tolerate higher light levels than their lower latitude counterparts (Frafjord 2021).

Despite the global distribution of bats, the only predators that specialize on bats are birds of prey found in the tropics (Fenton and Fleming 1976; Fenton et al. 1994). However, this does not mean that bats do not face predation elsewhere. There are records of mammals, fish, reptiles, amphibians, and birds occasionally preying on bats (Lima and O’Keefe 2013). This predation appears opportunistic in nature because flying bats are not easy to catch, and they are well protected in roosts during daytime. Avian predators, especially owls, are the most common threat to bats and can contribute 11% of their annual mortality (Speakman 1991). Owls share the same active period as bats, and opportunistic species or individuals can take bats quite regularly, with estimates at least 0.05% to 2% of their diet consisting of bats (Speakman 1991; Lesiński et al. 2009; Rosina and Shokhrin 2010). Specialized individuals residing close to bat roosts can constitute 21.9% to 36.5% of their diet on bats (Ignaczak et al. 2009). One such species predating on bats is the Tawny Owl (*Strix aluco*), a generalist that hunts opportunistically and may share the same roosts (e.g., old buildings) as bats (Speakman 1991; Solonen and Karhunen 2002; Ignaczak et al. 2009; Lesiński et al. 2009, 2012).

Predation on bats by owls is most severe when bats are abundant and relatively easy to catch, or when other prey are scarce (Roulin and Christe 2013). For instance, Lesiński et al. (2009) showed that a larger part of the diet of a Tawny Owl consists of bats in semi-urban areas. Because predation is opportunistic, roost emergence of bats can present predators with a rewarding opportunity. Bats tend to emerge every night after sunset from the same location, which means their presence is predictable in both time and space. Locations where large colonies emerge can be good feeding locations and territorial raptors have been observed waiting outside roosts before emergence begins (Rodriguez-Duran and Lewis 1985; Fenton et al. 1994).

Overall roost emergence is a high-risk situation for bats, and its timing could be connected to both predation (Jones and Rydell 1994; Russo et al. 2007; Thomas and Jacobs 2013; Arndt et al. 2018) and optimal foraging (Rydell et al. 1996). When conditions are lighter, earlier roost emergence provides longer foraging opportunities but may also increase predation risk. Hence the emergence time is potentially an antipredatory trade-off between effective foraging and avoidance of predators (Arndt et al. 2018). However, responses to controlled threats have not yet been observed in either emerging or foraging bats (Kalcounis and Brigham 1994; Zukal and Petrželková 2001; Baxter et al. 2006; Janos and Root 2014). Yet, bats have been observed delaying their emergence if the conditions require it, such as during exposure to loud music (Shirley et al. 2001) or heavy rainfall (Geipel et al. 2019). Free-flying, foraging bats should be able to avoid predators by switching locations (Fenton et al. 1994), but this may have a negative impact on nightly foraging because time and energy are lost while relocating to a potentially poorer feeding patch. Additionally, overall climatic conditions may affect both the emergence and foraging activity of bats (Catto et al. 1996; Frick et al. 2012)—especially conditions that deviate from the normal, e.g., heavy rain (Voigt et al. 2011) or low temperature (Gorman et al. 2021) may cause bats to decrease their activity or delay their emergence.

Nocturnal light levels depend on lunar phase, clouds, and latitude, and may also affect how bats respond to predator presence. At high northern latitudes, bats might have to adjust to both extreme weather conditions and light levels and prioritize their behavior differently than in southern locations. Summer nights in the boreal zone are short and not truly dark, which means that bats must both exit the roost at relatively high light conditions and may have to risk foraging in the presence of a visual predator during the few dark hours available each night (Rydell et al. 1996; Frafjord 2021).

In this study, we investigate whether bats display antipredatory behavior by delaying their emergence or by relocating while foraging, as determined by the timing/number of bat calls we detect. We hypothesize that the presence of an owl predator outside the roost will delay emergence in response to the calls of both adult and fledgling owls. However, if bats respond to all auditory disturbances (Shirley et al. 2001), we predict delayed emergence when either owl calls or rock music (here taken as a nonspecific stimulus) are played outside the roost. Furthermore, we predict that cold, rainy, and windy weather conditions will delay the emergence. Additionally, we hypothesize that bats decrease activity during nights when owl calls are played, compared to nights when either no treatment is applied or music is played. Alternatively, should bats avoid auditory disturbance in general during foraging, we predict reduced activity during nights with playback treatments (rock music, owls) compared to no-treatment control nights.

## Materials and methods.

### Data collection.

**Fig. 1.**
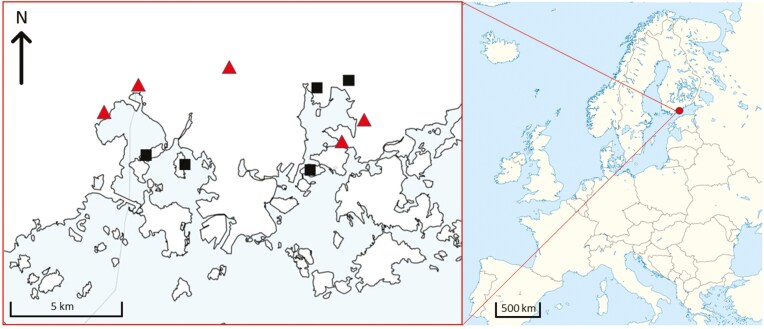
Sampling locations in the Helsinki area in southern Finland (latitude 61.2, WGS84). Locations marked with triangle were known Tawny Owl (*Strix aluco*) territories with nesting Tawny Owl observed at least once since 2017, locations marked with square had no Tawny Owl sightings (nesting included) since 2017. Data were collected from 30 July to 10 August 2020.

We studied the antipredatory responses of bats in 2 settings: 1) during roost emergence; and 2) during foraging. This study was conducted primarily in southern Finland with roost emergence sites between latitudes 60.0 and 61.9, with 1 site at latitude 63.5 (WGS84; Supplementary Data SD1) and foraging sites in Helsinki proximity (latitude 61.2, WGS84; Fig. 1). The chosen areas overlap with the distribution range of the Tawny Owl (*S. aluco*), that is known to occasionally feed on bats in Finland (Solonen and Karhunen 2002). Common bat species in the area are *Eptesicus nilssonii*, *Myotis brandtii*, *M. daubentonii*, *M. mystacinus*, *Plecotus auritus*, and *Pipistrellus nathusii*.

Bat species differ in their emergence timing, with diet and foraging strategy being main factors that impact the timing (see Jones and Rydell 1994). For the common species in Southern Finland species-specific emergence times after sunset based on literature are: *E. nilssonii*, 26 min (Jones and Rydell 1994); *M. daubentonii*, 84 min (Jones and Rydell 1994); *M. mystacinus*, 33 min (Jones and Rydell 1994); *P. auritus*, 44 min (Jones and Rydell 1994); and *P. nathusii* 11 to 50 min (Gelhaus and Zahn 2010). For *M. brandtii* we were not able to find a record of emergence time. Here, it is to be noted that some bat species emerge earlier, i.e., occasionally before sunset at high northerly latitudes (Michaelsen et al. 2018). However, to our knowledge, this has not been specifically studied with species that occur in Finland, with the exception of *E. nilssonii* which does emerge and forage in relatively light conditions in midsummer (Frafjord 2021). Bats echolocate in a species-specific manner in following frequencies: *E. nilssonii* at 30 kHz; *P. nathusii* at 40 kHz; and *P. auritus* at 29 kHz. All of the common *Myotis* species (*M. daubentonii*, *M. brandtii*, *M. mystacinus*) echolocate at 40 kHz and it is not possible to identify them to species level based on echolocation calls. Based on earlier studies, owls (inc. Tawny Owl) can hear sounds up to 21 kHz (Beason 2004) and thus unlikely can detect bats based on auditory detection. However, bats regularly vocalize and use social calls around 15 kHz while in their day roosts, so it is possible that an owl might be able to locate a roost using auditory detection.

In both roost emergence and foraging settings, we collected acoustic data on presence of bats by recording their echolocation calls with AudioMoth v.1.2.0 acoustic loggers (https://www.openacousticdevices.info/audiomoth). To test antipredatory responses, we used 2 controlled predation threats, and recorded Tawny Owl calls from both adults and fledglings because they might indicate the presence of an owl or an owl nest. Music (an opportunistic bat predator Ozzy Osbourne, under the graveyard, 2019) and silence were used as controls. Fledgling sounds were only used during roost emergence because the foraging data were collected toward the end of summer when the young could no longer be considered fledglings. The peak frequency of playback calls for adult tawny owls, fledglings, and music was 6.1 kHz, 12 kHz, and 5.5 kHz, respectively, as measured in Audacity (www.audacityteam.org). Although the hearing of bats is most sensitive around their species-specific echolocation range, they do hear sounds from at least 5 kHz and higher (Lattenkamp et al. 2021). We used a T-508 radio to play all playback treatments for a duration of 2 min with 20-s intervals concurrently with the bat call recording period at the roost exit and foraging grounds.

Emergence was recorded for 6 nights in June and 7 nights in July (8 to 13 June 2020 and 2 to 15 July 2020). In June, the emergence was recorded from 21:15 to 23:45. However, we noticed that this time window did not sufficiently catch the entire emergence, so for the July recording period the time was changed to 22:00 to 00:30. Our sampling consisted of 24 roosts, divided randomly in groups of 6 for 4 different treatments: 1) adult Tawny Owl; 2) fledgling Tawny Owl; 3) music; and 4) no treatment. For treatments 1 to 3, during the first 2 nights of both study periods emergence was recorded with no treatment sound broadcasted as to have an additional control to compare the treatment nights against. Because the playback treatments at each location were played through a loudspeaker, each roost required nightly attendance to turn the treatment audio on and off. Therefore, to maximize our data set, we used citizen scientists in data collection, with 2 roosts recorded by scientists and the rest by citizens. All citizens and scientists were responsible for a single location each. We set up a questionnaire online in spring 2020 to scout citizens with information of a roost location and gauge willingness to participate in the study. However, this approach meant that we had no prior knowledge of the species that were present at the roosts. We sent out 22 AudioMoths with instructions to ensure uniformity in data collection between citizens and scientists (Lundberg et al. 2021). AudioMoths and radios were placed 5 m from the roost entrance and 5 m away from each other and tied to a tree or a pole at chest height facing toward the roost. Ultimately, 1 device pair (AudioMoth and radio) was not used, resulting in 23 monitored roosts (6 roosts with the adult Tawny Owl treatment, 6 with fledgling Tawny Owl treatment, 5 with music treatment, and 6 with silent treatment).

Foraging data were collected from 30 July to 10 August 2020. We wanted to compare the behavior of bats under different assumed predation risk levels. For this, we chose 5 foraging areas with no known Tawny Owl presence (no sightings since 2017) and 5 areas with confirmed Tawny Owl presence (a nesting Tawny Owl at least once since 2017; Saurola 1987). The data of Tawny Owl sightings and nesting were acquired from BirdLife Finland, Tiira (https://www.tiira.fi/). Foraging areas were chosen based on their ecological qualities that bats are known to prefer while foraging, e.g., an opening by the forest. Altogether we had 10 foraging areas where acoustic data were recorded for 10 to 11 nights between 22:30 and 1:30. AudioMoths were fastened to a tree at a height of 2 m and faced toward open space. The radio was placed 5 m away from the AudioMoth on a rock (height of 10 to 50 cm) and faced toward the sky. For this data set, we rotated the treatments in a random order, so that within the 10- to 11-night recording period each site had 2 nights with adult Tawny Owl treatment and 2 nights with music. The rest of the nights were recorded with no treatment and treated as a silent control. All chosen areas had habitats that were semi-urban (e.g., recreation areas) and close to the treeline with either open space or an open flight path close by.

For weather variables, we used open-access weather data of the Finnish Meteorological Institute (https://www.ilmatieteenlaitos.fi/havaintojen-lataus#!/) and chose the weather station closest to each recording site. For each roosting site we used the weather conditions of the recording night at 22:00 (i.e., c. 1 h before the emergence). The used variables were temperature, sum of rain in 1 h and wind speed. We did not include weather variables in the foraging experiment model, because all the sites were situated in the Helsinki area with similar weather conditions and equal amounts of treatments were used during each night.

### Data analysis.

We used Kaleidoscope Pro (Wildlife Acoustics, Inc., 2020, v. 5.3.5) to analyze the acoustic data. We split the data into 10-s WAV files and screened for bats with the following signal detection parameters: frequency range between 16 and 120 kHz; length of detected pulses 1 to 200 ms; maximum inter-syllable gap 500 ms; and minimum number of detected pulses set to 1. To quantify bat activity, we went through the 10-s WAV files and gave each either the value of 1 if there were bats present in the file or 0 if there were no bats. These 10-s files did not necessarily represent individual bat passes but rather activity. All WAV files with or without bats were annotated with the location, recording date, recording night (1 to 13), recording time, and treatment.

We performed all statistical analyses using R software (version 4.2.1, R Core Team 2022). We used generalized linear mixed models (GLMMs) and analyzed the data from the emergence setting and foraging setting separately. For the emergence data, we tested if any of our treatments affected emergence time using Gaussian distribution and function lmer in packages “lme4” (Bates et al. 2015) and “lmerTest” (Kuznetsova et al. 2017). We transformed time into a continuous variable where 0:00 had a value of 0 and 23:59 had a value of 1 to remove the units of hours, minutes, and seconds. As our data were acoustic, and indicated bat presence and activity rather than individual bat passes, we used the time of the first observed bat in the evening as the start of the nightly emergence (see Jones and Rydell 1994). Other studies counting bats on site have used median emergence to control for individual outliers (Bullock et al. 1987). However, due to our datatype, the median emergence time would include considerably more inaccuracies (e.g., bats continuing to forage by roost entrance, returning bats) than the first observation. We did analyze the data set using median emergence (Supplementary Data SD2) but opted for the first observed bat in the final analysis for the reasons stated previously. Additionally, Jones and Rydell (1994) showed that median emergence and first emergence are significantly correlated. We attempted to separate the analysis by species, but this was not plausible because the data set included mixed colonies as well as species that only occurred once (Supplementary Data SD1) and would have been too small after exclusions. We had treatment (adult owl, fledgling, music, control) and the recording day of year (centered, both linear and quadratic effect) as fixed effects to capture the difference in daylight, but also in sampling effort. Treatments (adult owl, fledgling, music) were compared against the silent control (consisting of all silent control sites and silent control nights at the beginning of each period at treatment sites). We included temperature, rain, and wind variables to the model as fixed variables. Because all weather variables were available for 69 of 183 emergence cases, we ran separate models with weather variables (*n* = 69) and without weather variables (*n* = 183). Site and recording night were treated as random variables in both models, because bat activity may differ between sampling sites due to, e.g., different species or environmental variables.

For foraging data, we used GLMMs with a binomial distribution (function glmer in “lme4”; Bates et al. 2015) because a 10-s segment within the study period could either contain bat calls (1) or not (0). The data were aggregated on the night level (number of occurrences and absences). The occurrence of bats was explained by 5 models with different fixed variables: (1) no fixed variables; (2) owl presence or absence (territory); (3) owl or music playback used or not (treatment); (4) treatment + territory; and (5) treatment * territory. All 5 models had the same random variables: site and recording night. We did not include weather variables to the analyses as the sites were so close to each other that they would have had data from the same weather station. Furthermore, the weather was stable throughout the recording period and there was no rain or high wind speed. The daily weather and other unknown daily variation in conditions are captured by the recording night random factor. We ranked models based on the AICc values of the models using package “MuMIn” in R (Bartoń 2020). We considered *P*-values < 0.05 significant for all tests. We tested the collinearity of variables for the emergence data set and foraging data set using function vif of the “usdm” package (Naimi et al. 2013) and did not find signs of strong collinearity (all variation inflation factor [VIF] < 1.41).

## Results

### Roost sampling.

The average first emergence time of bats with the transformed values was 0.965 (Table 1), and transformed back to 24-h time stands for 23:16 (min 21:15 to max 00:16; SD = 38 min, *n* = 183 emergence times). Altogether we had 17,409 10-s files with bat activity. We did not specifically separate bat calls by species but took note of the species composition at each roost (Supplementary Data SD1). Most common species and species compositions were *E. nilssonii* (*n* = 5) and *Myotis* spp. (*n* = 7) and the combination of these species (*n* = 8). Additionally, we had roosts that consisted of *Myotis* spp. and *P. nathusii* (*n* = 2) and *P. auritus* (*n* = 1). The adult owl treatment group had the most diverse species composition (3 *E. nilssonii *+ *Myotis* spp. roosts, 1 *E. nilssonii* roost, 1 *Myotis* spp. roost, 1 *P. auritus* roost) followed by the silent control group (3 *E. nilssonii* + *Myotis* spp. roosts, 1 *E. nilssonii* roost, 1 *Myotis* spp. roost, 1 *Myotis* spp. + *P. nathusii* roost). The fledgling treatment group consisted mostly of *Myotis* spp. (4 *Myotis* spp. roosts, 1 *E. nilssonii* + *Myotis* spp. roost, 1 *Myotis* spp. +* P. nathusii* roost) and music treatment group mostly of *E. nilssonii* (3 *E. nilssonii* roosts, 1 *Myotis* spp. roost, 1 *E. nilssonii* + *Myotis* spp. roost).

**Table 1.** Coefficient estimates and test values of the model explaining the emergence timing of bats. Both models with (*n* = 69 emergence cases) and without (*n* = 183) weather variables are shown. Treatment included sounds of music (music), fledgling tawny owls (fledgling), and adult tawny owls (owl) compared to the control treatment (control treatment group and silent control nights of all sites). The day of the year (DoY) had both linear and quadratic effect. Significant variables (*P* < 0.05) are in bold, and tendencies (*P* < 0.1) are shown in italic.

Variable	Estimate	SE	df	*t*	*P*
Without weather variables					
Intercept	**0.965**	**0.005**	**28.9**	**205.3**	**<0.001**
Treatment, music	−0.006	0.006	93.0	−1.0	0.325
Treatment, fledgling	−0.003	0.006	81.4	−0.5	0.645
Treatment, owl	*0.011*	*0.006*	*113.1*	*1.8*	*0.074*
DoY, linear	0.015	0.045	26.6	0.3	0.736
DoY, quadratic	−0.019	0.058	22.8	−0.3	0.749
With weather variables					
Intercept	**1.019**	**0.022**	**39.8**	**46.7**	**<0.001**
Treatment, music	−0.006	0.009	53.6	−0.7	0.478
Treatment, fledgling	−0.006	0.009	54.9	−0.7	0.500
Treatment, owl	0.004	0.010	57.2	0.4	0.730
DoY, linear	−0.120	0.132	8.1	−0.9	0.392
DoY, quadratic	−0.118	0.260	7.7	−0.5	0.662
Temperature	**−0.005**	**0.002**	**48.8**	**−3.3**	**0.002**
Rain	0.003	0.009	60.0	0.4	0.712
Wind	**0.007**	**0.003**	**60.8**	**2.1**	**0.040**

**Fig. 2.**
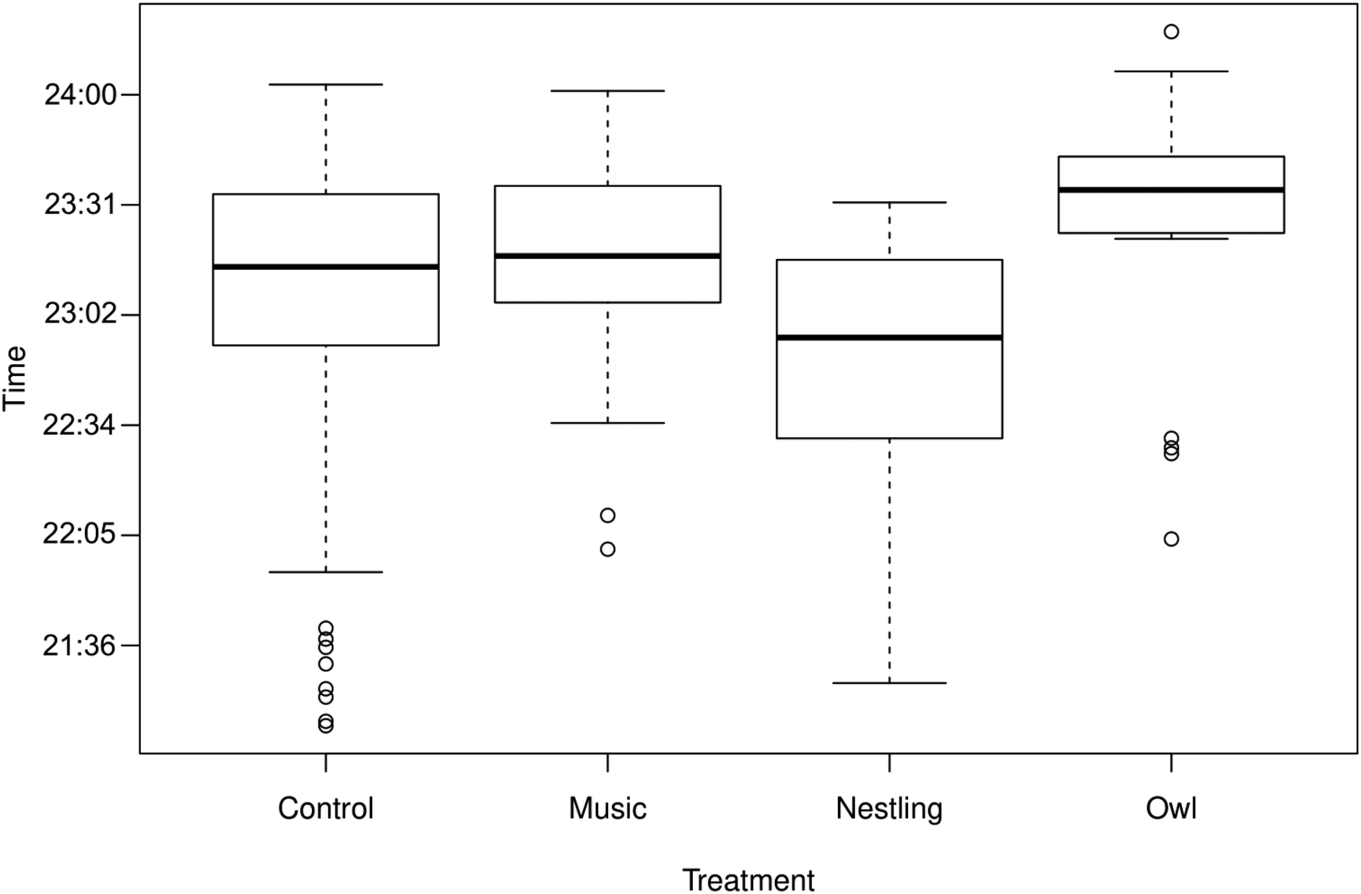
Observed values of emergence times of bats from their roosting sites under different treatments: control (silent treatment group and silent control nights from all sites); music (nonspecific stimulus); fledgling (fledgling Tawny Owl, *Strix aluco*); and owl (adult Tawny Owl). Emergence behavior was recorded in 23 roosts. Bats showed a tendency (*P *= 0.074) to delay their emergence by approximately 16 min while under owl treatment compared to control treatments. Bold line shows the median of the observations. Box gives the limits of 25% and 75% of the observations and whiskers are the rest of the observations. Open dots are observations that are farther than 2 times from the broadness of the box.

**Fig. 3.**
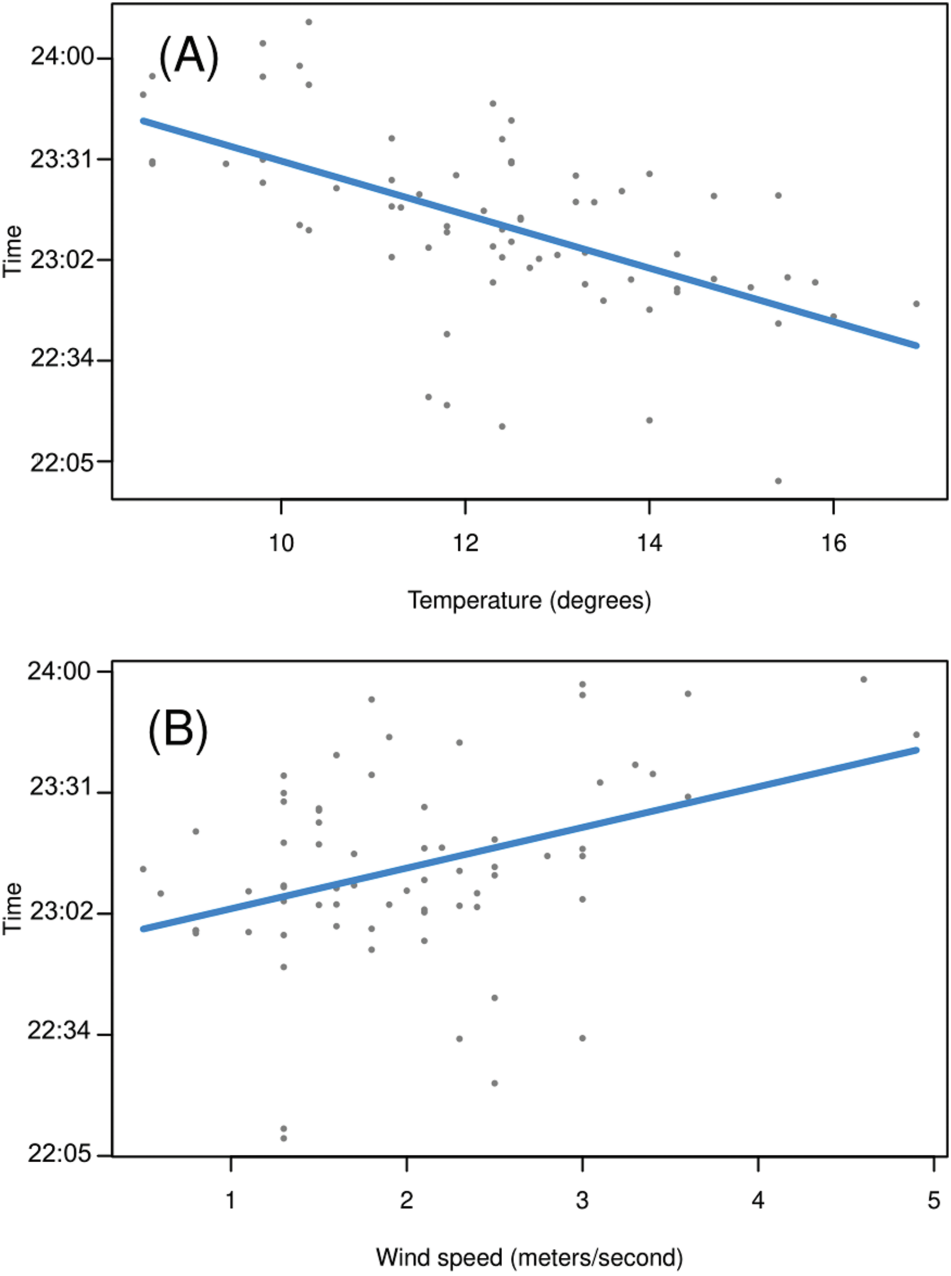
Emergence times of bats in relation to weather variables: (A) bat emergence times were delayed with decreasing temperature (°C); and (B) increasing wind speed (m/s).

In the model with larger sample size without weather variables the first bats showed a tendency to leave the roost site on average 16 min (*b* = 0.0113 ± 0.0062, 0.0113 * 24 h * 60 min/h = 16 min) later when the recordings of adult Tawny Owl were played compared to the silent control (*P* = 0.074; Table 1; Fig. 2). There was no response to the playback treatments of fledgling Tawny Owl or music (respectively, *P *= 0.645 and *P *= 0.325; Table 1; Fig. 2). The model with the weather variables, but with lower sample size, did not detect any response of the treatments, but colder and windier weather significantly delayed emergence times (Table 1; Fig. 3).

### Foraging sampling.

Altogether we had 21,304 10-s files with bat activity. The most common species were *E. nilssonii* and *Myotis* spp., but we also had observations of *P. nathusii*.

**Fig. 4.**
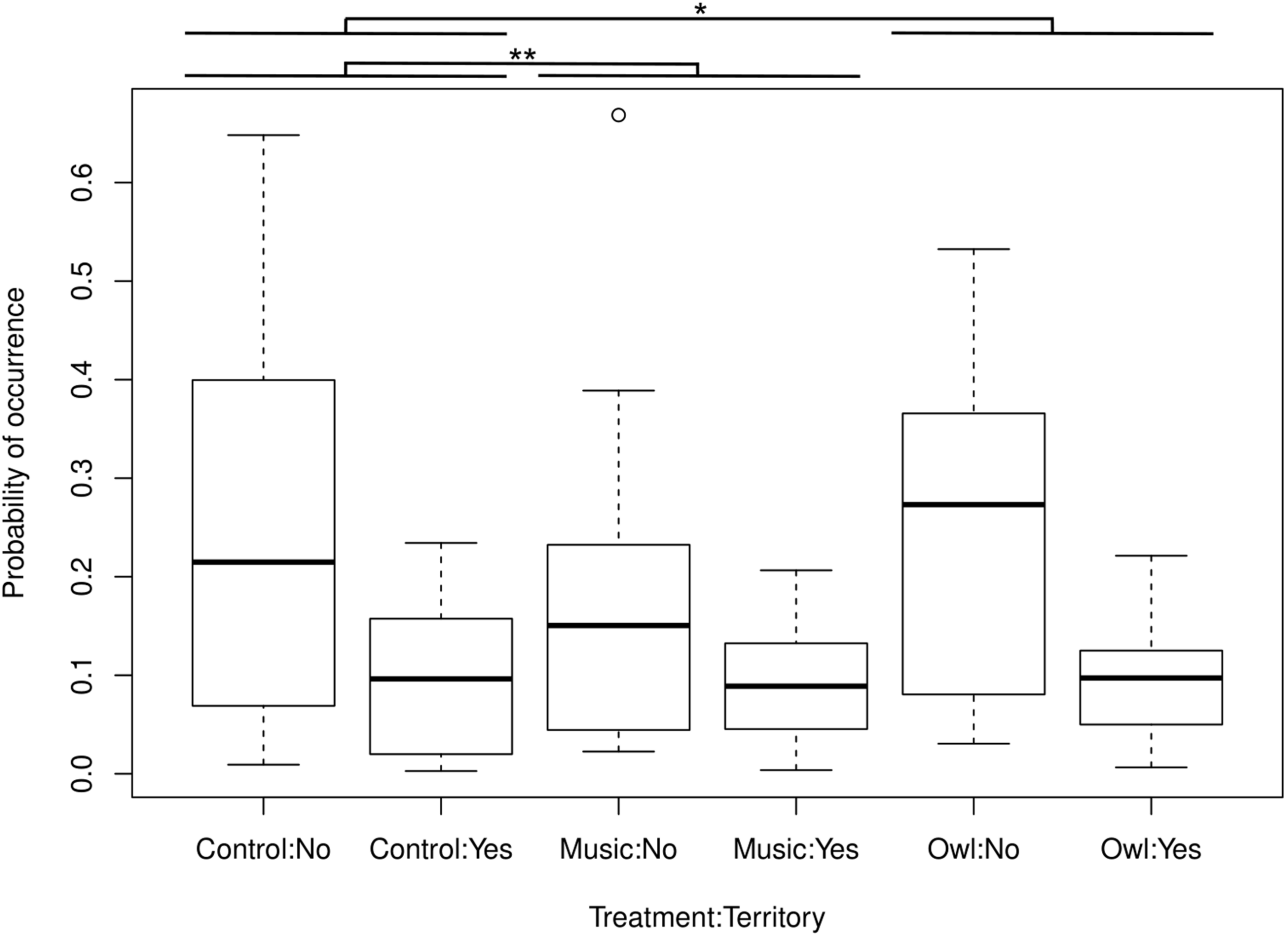
Probability of occurrence in foraging bats under different treatments based on observed values: control (no treatment), music (nonspecific stimulus), and owl (adult Tawny Owl, *Strix aluco*) within sites with no Tawny Owl presence (No) and known Tawny Owl territories (Yes). The data were collected in 10 sites that had the treatments rotated through a 10- to 11-night recording period (owl treatment = 2 nights/site, music treatment = 2 nights/site, control = 6 to 7 nights/site). Bat occurrence was negatively influenced by music and owl treatments within owl territories. Bold line shows the median of the observations. Box gives the limits of 25% and 75% of the observations and whiskers are the rest of the observations. Open dots are observations that are farther than 2 times from the broadness of the box.

Playback treatments of adult tawny owls and music negatively influenced bat activity, but only within and in combination with owl territories (respectively, *P *= 0.015 and *P *= 0.002; Table 2; Fig. 4). Alone, neither territory (*P *= 0.137; Table 2) nor treatment (owl *P *= 0.255, music *P* = 0.980; Table 2) had any effect. Bat activity within non-territories was not affected by owl calls or music. The model including both treatment and owl territory as well as their interaction was clearly top-ranked (∆AICc > 8; Table 3).

**Table 2.** Coefficient estimates and test values of the model explaining the occurrence of foraging by bats. Treatment included sounds of music and adult tawny owls. Significant variables (*P* < 0.05) are in bold.

Variable	Estimate	SE	*z*	*P*
Intercept	**−1.548**	**0.501**	**−3.09**	**0.002**
Treatment, music	−0.001	0.032	−0.03	0.980
Treatment, owl	0.031	0.026	1.14	0.255
Territory	−1.097	0.737	−1.49	0.137
Treatment, music: territory	**−0.157**	**0.050**	**−3.13**	**0.002**
Treatment, owl: territory	**−0.118**	**0.048**	**−2.44**	**0.015**

**Table 3.** Ranking of models explaining occurrences of foraging bats using AICc. Number of model parameters (*K*), AIC difference to the top-ranked model (∆AICc), and AIC weights are shown. Territory is whether the site had owl territory or not and treatment is whether sound treatments were used or not.

Model	*K*	∆AICc	Weight
Territory * treatment	8	0.00	0.956
Treatment	5	8.40	0.014
Territory + treatment	6	8.45	0.014
Territory	4	9.52	0.008
Null model	3	9.57	0.008

## Discussion

Our results show that bats delay their emergence based on prevailing weather conditions, specifically cold temperatures and high wind speed. Overlooking the influence of weather, bats showed a nonsignificant tendency (*P* = 0.074; Table 1) to delay their emergence by 16 min on average while under simulated predation threat, the playback vocalization of adult Tawny Owl (Fig. 2). The vocalization of Tawny Owl fledglings did not impact emergence time, so it would seem that bats did not connect fledgling calls to either predation threat or presence of adult owls. Although known as an artist that opportunistically preys on bats, the playback treatment of Ozzy Osbourne music did not affect roost exit times. As none of our playback treatments produced statistically significant delay in emergence, our original hypothesis of antipredatory behavior was rejected. Despite there being multiple studies suggesting that bats alter their emergence time based on predation threat (Russo et al. 2007; Thomas and Jacobs 2013; Arndt et al. 2018), no studies with a simulated threat have produced significant results (Kalcounis and Brigham 1994). However, previous studies have focused on a single colony (Kalcounis and Brigham 1994; Zukal and Petrželková 2001), but here we put forth the largest data set (23 roosts monitored simultaneously) collected for an antipredator response experiment on emerging bats. However, it is possible that the data set is still too small to show any possible significant results, and only being able to capture possible tendencies of antipredatory behavior.

We recognize that our method of monitoring emergence with acoustic devices, while maximizing data, leaves room for error. It is possible that a bat continued to forage close to the roost exit after emergence or that a foraging bat from another roost passed by the detector before the actual emergence began. However, we assume that these would bias the results in a similar manner across all treatments and sites. Therefore, we do not think that our data collecting method would have caused false results, although we acknowledge that, for instance, differences in the environment may have affected the results. Furthermore, we were not able to take species into account in the analysis, which can add more stochasticity to the results as different species have different emergence times. No treatment was represented by a single species in the final data set, but it is possible that the results could be explained by species-specific differences and not antipredatory or weather-dependent behavior. Adult owl and silent control treatment groups were represented by complementary species compositions (*E. nilssonii*, *Myotis* spp., *P. auritus*, and mixed colonies of *E. nilssonii* + *Myotis* spp., and *Myotis* spp. + *P. nathusii*), but fledgling owl treatment consisted mostly of *Myotis* spp. and music treatment group mostly of *E. nilssonii*. This could cause the fledgling owl treatment group to emerge later than average and music treatment group to emerge earlier than average. However, both groups emerged a few minutes earlier than the silent control group, but the results were not statistically significant (respectively, *P *= 0.645 and *P *= 0.325; Table 1). However, it is possible that the music treatment group delayed their emergence in response to treatment, but it was left unnoticed due to the species composition and species-specific behavior of earlier emergence.

We also presented a hypothesis that if foraging bats expressed antipredatory responses, then we would see a decrease in bat activity while Tawny Owl calls were played, but not on nights when music or no sound were broadcasted. Our hypothesis was not supported because bats decreased their activity both on nights when owl sounds and music were played, supporting the hypothesis that bats react to noise in general. However, this was only the case when bats were foraging in known Tawny Owl territories. The results are similar to Baxter et al. (2006) who detected decreased bat activity during owl calls and control sounds suggesting that bats react to general auditory disturbance. Another similar study did not find a difference in activity with regards to treatment (Janos and Root 2014), much like how bats reacted in our study in the areas with no known owl activity. Similarly, bats have been found to decrease their activity (Finch et al. 2020) or avoid areas (Schaub et al. 2008) because of traffic noise. However, in our study the noise aversion was only present at Tawny Owl territories, so it might be possible that the bats were aware of heightened threat levels and were therefore more vigilant and responsive to any auditory cues (Laundré et al. 2010). Bats have been found to be more inefficient and inaccurate when either foraging (Allen et al. 2021) or drinking (Domer et al. 2021) under noise disturbance, and we suspect that bats cannot afford this inefficiency in high-risk areas. However, here it is only speculation and calls for further studies. Additionally, it is possible that our study sites differed in other significant ways than being owl territories.

The addition of climatic variables to our models revealed that weather explained more of the variation in time of departure from the roost than our predator treatments. However, these results are ambiguous because due to a lack of data, we were able to test the effect of climatic variables for only a portion of our sampling events. Additionally, we were not able to confidently estimate the effect of climatic variables in the model for predator avoidance during foraging due to a lack of fine-scale variability in the available weather data, i.e., an owl territory and a control site may have fallen within the same coverage area with regards to weather observations. Climatic variables are known to influence time of departure from roosts in bats (Frick et al. 2012), but surprisingly, unlike wind and temperature, rain did not have a significant impact on time of departure in our study despite its recognized negative impact on flight performance (Voigt et al. 2011). Overall, our results indicate that bats may be responding to the most acute factor contributing to their survival at any given time, be it the weather or predators.

Through our study, we discovered that bats have the potential to shift their roost emergence and possibly present antipredatory behavior and limit their activity in a high-risk area (owl territory). To explain our results, we must ask why possible antipredatory responses are not more readily observed. Animals have limited time and energy, which is why they must optimize how they divide these during their daily activities (Lima and Bednekoff 1999; Cresswell 2008). The time and energy spent avoiding predators is time subtracted from other activities, for instance, foraging. We suggest that bats may be able to differentiate situational risk levels and allocate their behavior accordingly. The ability to exhibit antipredatory behavior only in high-risk situations would be especially important to boreal bats that must operate and forage within the limited hours of short northern summer nights. Most likely bats cannot afford to lose limited foraging time during the short boreal summer but need to avoid predators as effectively as possible as well as consider other environmental threats. Here, to maximize their survival (i.e., avoid predation and gain energy), these actions should be balanced, and the optimal course of action might be to begin and continue to forage when the likelihood of a successful attack is reduced and display antipredatory behavior when a successful attack is more probable, i.e., within an established owl territory or while emerging from the roost. Whether these actions are effective as predator avoidance is outside of the scope of this study.

It has been argued that boreal bats are not under predation pressure or have not been exposed to nocturnal predation (Karlsson et al. 2002). This argument is based on the observed lack of lunar phobia in boreal bats, unlike their tropical counterparts (Karlsson et al. 2002; Roeleke et al. 2018). However, our results indicate a possibility of antipredatory responses, suggesting that boreal bats might be under predation pressure. We argue that because ambient lighting conditions vary greatly across latitudes, bats and nocturnal animals in general would have to adapt to the lack of complete darkness and optimize their predator avoidance while possibly being aware of existing threat levels. Furthermore, with climatic conditions influencing our results to such a high degree, a more holistic understanding of decision-making in bat behavior is needed. To conclude, we are aware that this study does not clearly demonstrate antipredatory responses in bats, but rather introduces the possibility and calls for further antipredatory studies in which possible existing predation threat is taken into consideration.

## Supplementary data

Supplementary data are available at *Journal of Mammalogy* online.

**Supplementary Data SD1.** The treatments, municipalities in Finland, latitudes, and species of the roosts observed for the roost emergence study.

**Supplementary Data SD2.** Coefficient estimates and test values of the model explaining the emergence timing of bats using median time of emergence.

gyae124_suppl_Supplementary_Datas_SD1

gyae124_suppl_Supplementary_Datas_SD2

## Data Availability

The data used and the R code will be deposited into GitHub prior to publication.
