# Supplementary material for: Antipredator responses of bats during short boreal nights with variable climatic conditions
Source: J Mammal. 2024 Oct 29;106(2):385–93. doi: 10.1093/jmammal/gyae124 (PMC11933277; doi:10.1093/jmammal/gyae124)
Supplement: gyae124_suppl_Supplementary_Datas_SD1 [file gyae124_suppl_supplementary_datas_sd1.docx]

**Supplementary Data SD1.** The treatments, municipalities in Finland, latitudes (WGS84) and species of the roosts observed for the roost emergence study.

| Treatment | Municipality | Latitude, N | Bat species |
| --- | --- | --- | --- |
| adult owl 1 | Riihimäki | 60.7 | *Plecotus auritus* |
| adult owl 2 | Pyhtää | 60.5 | *Eptesicus nilssonii, Myotis* sp |
| adult owl 3 | Laitila | 60.9 | *Eptesicus nilssonii, Myotis* sp |
| adult owl 4 | Orivesi | 61.7 | *Myotis* sp |
| adult owl 5 | Turku | 60.5 | *Eptesicus nilssonii* |
| adult owl 6 | Inkoo | 60.0 | *Eptesicus nilssonii, Myotis* sp |
| fledgling owl 1 | Kouvola | 60.9 | *Myotis* sp |
| fledgling owl 2 | Nurmes | 63.5 | *Eptesicus nilssonii, Myotis* sp |
| fledgling owl 3 | Tenhola | 60.1 | *Myotis* sp |
| fledgling owl 4 | Turku | 60.5 | *Myotis* sp |
| fledgling owl 5 | Havumäki | 61.9 | *Myotis* sp |
| fledgling owl 6 | Kirkkonummi | 60.1 | *Myotis* sp, *Pipistrellus nathusii* |
| control, music 1 | Espoo | 60.2 | *Eptesicus nilssonii* |
| control, music 2 | Sammatti | 60.3 | *Eptesicus nilssonii* |
| control, music 3 | Porvoo | 60.4 | *Myotis* sp |
| control, music 4 | Lempäälä | 61.3 | *Eptesicus nilssonii* |
| control, music 5 | Salo | 60.4 | *Eptesicus nilssonii, Myotis* sp |
| control, silence 1 | Kuhmoinen | 61.6 | *Myotis* sp, *Pipistrellus nathusii* |
| control, silence 2 | Kirkkonummi | 60.1 | *Myotis* sp |
| control, silence 3 | Köyliö | 61.1 | *Eptesicus nilssonii* |
| control, silence 4 | Lahti | 61.0 | *Eptesicus nilssonii, Myotis* sp |
| control, silence 5 | Siuntio | 60.1 | *Eptesicus nilssonii, Myotis* sp |
| control, silence 6 | Vainikkala | 60.9 | *Eptesicus nilssonii, Myotis* sp |
