# Supplementary material for: Antipredator responses of bats during short boreal nights with variable climatic conditions
Source: J Mammal. 2024 Oct 29;106(2):385–93. doi: 10.1093/jmammal/gyae124 (PMC11933277; doi:10.1093/jmammal/gyae124)
Supplement: gyae124_suppl_Supplementary_Datas_SD2 [file gyae124_suppl_supplementary_datas_sd2.docx]

**Supplementary Data** **SD2**. Coefficient estimates and test values of the model explaining the emergence timing of bats using median time of emergence. Both models with (n = 69 emergence cases) and without weather variables (n = 183) are shown. Significant variables (*P* < 0.05) are bolded and tendencies (*P* < 0.1) are shown in italic.

| Variable | Estimate | SE | d.f. | *t* | *P* |
| --- | --- | --- | --- | --- | --- |
| Without weather variables |  |  |  |  |  |
| Intercept | **0.983** | **0.003** | **28.6** | **280.9** | **<0.001** |
| Treatment, fledgling | -0.004 | 0.004 | 137.6 | -0.9 | 0.3905 |
| Treatment, music | -0.002 | 0.004 | 145.3 | -0.4 | 0.709 |
| Treatment, owl | *0.009* | *0.005* | *147.0* | *1.9* | *0.053* |
| DoY, linear | 0.016 | 0.024 | 28.5 | 0.7 | 0.511 |
| DoY, quadratic | -0.003 | 0.030 | 20.9 | -0.1 | 0.931 |
| With weather variables |  |  |  |  |  |
| Intercept | **0.985** | **0.011** | **49.1** | **89.8** | **<0.001** |
| Treatment, fledgling | **-0.012** | **0.005** | **51.8** | **-2.5** | **0.014** |
| Treatment, music | -0.003 | 0.005 | 52.7 | -0.6 | 0.565 |
| Treatment, owl | *0.009* | *0.005* | *48.7* | *1.8* | *0.085* |
| DoY, linear | -0.049 | 0.031 | 10.6 | -1.6 | 0.142 |
| DoY, quadratic | -0.086 | 0.058 | 8.7 | -1.5 | 0.173 |
| Rain | **0.012** | **0.004** | **54.0** | **2.7** | **0.010** |
| Temperature | 0.001 | 0.001 | 56.3 | 0.4 | 0.716 |
| Wind | 0.001 | 0.002 | 59.2 | 0.1 | 0.882 |
